# Patient and doctor attitudes and beliefs concerning perioperative do not resuscitate orders: anesthesiologists’ growing compliance with patient autonomy and self determination guidelines

**DOI:** 10.1186/1471-2253-13-2

**Published:** 2013-01-15

**Authors:** Christopher M Burkle, Keith M Swetz, Matthew H Armstrong, Mark T Keegan

**Affiliations:** 1 Department of Anesthesiology, Mayo Clinic, 200 First Street SW, Rochester, MN, USA; 2 Department of Medicine, Mayo Clinic, 200 First Street SW, Rochester, MN, USA

**Keywords:** Do not resuscitate, Perioperative medicine, Patient decisions/choice

## Abstract

**Background:**

In 1993, the American Society of Anesthesiologists (ASA) published guidelines stating that automatic perioperative suspension of Do Not Resuscitate (DNR) orders conflicts with patients’ rights to self-determination. Almost 20 years later, we aimed to explore both patient and doctor views concerning perioperative DNR status.

**Methods:**

Five-hundred consecutive patients visiting our preoperative evaluation clinic were surveyed and asked whether they had made decisions regarding resuscitation and to rate their agreement with several statements concerning perioperative resuscitation. Anesthesiologists, surgeons and internists at our tertiary referral institution were also surveyed. They were asked to assess their likelihood of following a hypothetical patient’s DNR status and to rate their level of agreement with a series of non-scenario related statements concerning ethical and practical aspects of perioperative resuscitation.

**Results:**

Over half of patients (57%) agreed that pre-existing DNR requests should be suspended while undergoing a surgical procedure under anesthesia, but 92% believed a discussion between the doctor and patient regarding perioperative resuscitation plans should still occur. Thirty percent of doctors completing the survey believed that DNR orders should automatically be suspended intraoperatively. Anesthesiologists (18%) were significantly less likely to suspend DNR orders than surgeons (38%) or internists (34%) (p < 0.01).

**Conclusions:**

Although many patients agree that their DNR orders should be suspended for their operation, they expect a discussion regarding the performance and nature of perioperative resuscitation. In contrast to previous studies, anesthesiologists were least likely to automatically suspend a DNR order.

## Background

It is reported that up to 15% of patients undergoing surgery have pre-existing DNR orders on record. First described in 1960 to treat witnessed intraoperative events, closed cardiac massage became increasingly utilized to treat all patients suffering cardiac arrest [1-3]. In 1983, the President’s Commission for the Study of Ethical Problems in Medicine suggested favoring implicit consent to CPR, thereby requiring an order for resuscitation to be withheld [1]. In 1988, the Joint Commission on Accreditation of Healthcare Organizations required that hospitals develop policies on resuscitative efforts [3,4]. Following passage of the Patient Self-Determination Act in 1991, hospitals receiving Medicare funds were required to provide patients with written information regarding their right to make health care decisions, including advance directives [5,6].

The surgical setting has been described as “the last bastion of resistance to acceptance of DNR orders [7].” The operating room (OR) environment offers challenges to following pre-existing DNR orders [3,8]. OR personnel - particularly anesthesia providers - are trained and equipped to provide resuscitation efforts. Limiting this skill causes discomfort to some providers [1,9]. Furthermore, resuscitation in this monitored and controlled environment is often more successful and providers may find it difficult not to intervene while a patient physiologically deteriorates [5,10-15].

Despite potentially conflicting goals and interests, decisions involving perioperative resuscitation remain largely patient choices. In 1993, the American Society of Anesthesiologists (ASA) published guidelines stating that automatic suspension of DNR orders infringed upon a patient’s right to self-determination [16]. The 2001 ASA guidelines (reaffirmed in 2008) recapitulated this concern [17]. The American College of Surgeons and the Association of Operating Room Nurses have expressed similar sentiments [16,18,19].

Given that several professional guidelines have been in place for nearly 20 years, we explored both patient and doctor views concerning perioperative DNR status.

## Methods

### Patient assessment

Following Mayo Clinic Institutional Review Board approval, 500 sequential patients assessed in the preoperative evaluation clinic at our tertiary referral center in January 2012 were invited to voluntarily complete a survey (shown in Additional file: 1). Informed consent was obtained from each study patient prior to administration of the survey. Respondents were asked if they had discussed and/or documented decisions regarding resuscitation. Questions concerning ethical, philosophical and practical aspects of perioperative resuscitation were posed and respondents rated their agreement on a 5-point scale. Patient age was also recorded.

### Doctor assessment

In May 2011, anesthesiologists, surgeons and internists (collectively “doctors” throughout text) at our institution were asked via e-mail (and subsequent reminders) to complete an anonymous on-line survey relating to perioperative DNR orders. In compliance with Mayo Clinic Institutional Review Board requirements, informed consent was obtained from each doctor prior to their completion of the survey. Surveys were managed using a secure web application (REDCap, Vanderbilt University, Nashville, TN). The survey questions and responses are shown in Additional file: 2. Respondents were surveyed regarding agreement with a generic statement regarding automatic suspension of perioperative DNR orders. Next, a scenario was presented in which a hypothetical patient had a DNR order in place. Respondents rated their likelihood of following the hypothetical patient’s DNR status on a 5-point scale. Participants were asked to rate their agreement with statements exploring the impact of patient condition at time of surgery, age of DNR order, lack of preoperative discussion and fear of legal liability had on doctors’ decisions to respect (or not) the DNR order. Agreement with the scenario-related statements was rated on a 5-point scale. Lastly, respondents rated their agreement with a series of statements concerning ethical, philosophical and practical aspects of perioperative resuscitation on a 5-point scale.

### Statistical analyses

Responses of “prefer not to answer” were treated as missing data. Data were analyzed using descriptive statistics, the Cochran Armitage trend test and Chi-square and Fisher exact test analyses, as appropriate. For the Cochran Armitage trend test analyses, responses indicating the strength of agreement or disagreement with statements were treated as ordinal data. Responses to Questions 3–7 of the Patient Survey and 4–14 of the Doctor Survey were also grouped into two levels, depending on the question: [Very/somewhat likely (“Likely”) and very/somewhat unlikely (“Unlikely”); Very/somewhat important (“Important”) and very/somewhat unimportant (“Unimportant”); Strongly agree/agree (“Agree”) and Strongly disagree/disagree (“Disagree”)]and treated as categorical data for Chi-square and Fisher exact test analyses, as appropriate, to allow for more simple comparisons and improve clarity of data presentation. A *P*-value of less than 0.05 was considered significant.

## Results

### Patient assessment

The overall patient response rate was 84% (418/500). Seventy-seven percent reported that they had made a decision regarding resuscitation if they fell seriously ill. Nevertheless, only 54% of preferences were documented in writing. Eighty-five percent of respondents were 50 years of age and older. Decisions on resuscitation were more common in older patients (79%) than younger patients (66%) (p = 0.03) and older patients were more likely to have documented their decision (older 57%, younger 26%, p < 0.01).

More than half (57%) agreed that pre-existing requests not to be resuscitated should be suspended while undergoing a surgical procedure under anesthesia (Patient Question 3), but 92% believed discussions between doctors and patients regarding perioperative resuscitation plans should always occur (Patient Question 4) (Table 1). One-third of respondents *strongly* disagreed that because of the complexity of the surgical environment, decisions concerning resuscitation should be left to anesthesiologists and surgeons alone (Patient Question 6). Further Patient Survey results are provided in Table 1.

**Table 1:** Patient responses to a series of general statements

	**Agree**		**Neither agree or disagree**	**Disagree**	
	**Strongly**	**Some-what**		**Some-what**	**Strongly**
Preoperative DNR requests should be suspended for surgical procedures	131 (32%)	104 (25%)	62 (15%)	40 (10%)	58 (14%)
Requests not to be resuscitated should always be discussed between patient and surgeon or anesthesiologist	309 (74%)	74 (18%)	17 (4%)	5 (1%)	4 (<1%)
Decisions about intraoperative resuscitation should be left up to surgeons and anesthesiologists alone because patients cannot fully understand the complexities involved with a surgical process	87 (21%)	94 (23%)	28 (7%)	54 (13%)	137 (33%)
The type of surgical procedure should influence whether a patient’s request not to be resuscitated is followed	113 (28%)	115 (28%)	61 (15%)	32 (8%)	75 (18%)
If a patient’s request to forgo resuscitation is suspended for a surgical procedure, it should be reinstated at a predetermined point following anesthesia recovery.	206 (50%)	120 (29%)	50 (12%)	10 (2%)	8 (2%)

### Doctor assessment

We received 384 completed physician surveys. The response rates were 53% (101 of 109), 22% (109 of 491), and 15% (157 of 1074) for anesthesiologists, surgeons and internists respectively (Table 2). Of the 384 surveys returned, 26% were returned by anesthesiologists, 28% by surgeons, 41% by internists and 4% “other” (Table 2).

**Table 2:** Doctor Demographics

**Practice Type**	**Surgery**	**Internal Medicine Focus**			**Anesthesiology**	**Other**
	109 (28%)	General		Subspecialty		
		66 (17%)		91 (24%)	101 (26%)	16 (4%)
Years in Practice	0-3 yrs	4-7 yrs	8-15 yrs	≥ 16 yrs	Fellow	Resident
	27 (7%)	34 (9%)	56 (15%)	120 (31%)	57 (15%)	89 (23%)

Thirty percent believed that DNR orders should automatically be suspended intraoperatively (Doctor Question 3), 52% disagreed with this practice and 17% were unsure. Anesthesiologists (18%) were significantly less likely to unilaterally suspend DNR orders than surgeons (38%) or internists (34%) (p < 0.01). Number of years in practice had no significant impact on whether respondents followed DNR orders. (p = 0.28)

### Scenario-based assessment

Fifty-four percent of doctors reported that they were unlikely to follow the patient’s DNR request in the scenario presented while 28% were likely to comply with the DNR and 18% were unsure. That the DNR order was 5 years old was important to 64% of those who would suspend the DNR order and to 74% of those who would follow the order. (p = 0.13) Fear of legal liability for their decision was important to 55% and 66% of the suspending and following groups, respectively (p = 0.11). Lack of a preoperative discussion between patient and doctor was important to 93% of those suspending and to 86% of those following the DNR order. (p < 0.01)

### General questions assessment

Over half (55%) agreed that DNR requests were illogical during surgical procedures (anesthesiologists 54%, surgeons 75%, internists 52%, p < 0.11) (Doctor Question 8) (Table 3). Of those expressing a preference, 77% agreed that anesthesia teams should be permitted to use all their skills regardless of DNR status (Doctor Question 9). Surgeons (89%) were more likely to agree than either anesthesiologists (67%) or internists (77%). (p < 0.01) Despite these responses, 76% of doctors surveyed disagreed that patients could not appreciate the idiosyncrasies of surgical care enough to formulate decisions about resuscitation efforts (Doctor Question 10). This response was independent of specialty (p = 0.76). Half of all doctor respondents agreed with the statement that intraoperative DNR orders should be respected because resuscitative issues are based on a patient’s value system rather than doctor preferences (Doctor Question 11). Excluding those who neither agreed nor disagreed, this figure increased to 61%. Surgeons (37%) were less likely to agree than the other specialties p < 0.01). Over three-quarters of respondents agreed that patients capable of consenting to surgery could separately determine their desire for perioperative resuscitation. However, fewer surgeons agreed (75%) than either anesthesiologists (91%) or internists (89%), (p < 0.01).

**Table 3:** Doctor responses to a series of general statements

	**Agree**		**Neither agree or disagree**	**Disagree**	
	**Strongly**	**Some-what**		**Some-what**	**Strongly**
Since anesthesia for surgery basically represents a resuscitative effort (endotracheal intubation, pressor support, etc.), DNR (“Do not Resuscitate”) status makes no logical sense in the context of a surgical procedure requiring an anesthetic.	87 (23%)	123 (32%)	26 (7%)	102 (27%)	44 (12%)
The delivery of every anesthetic likely involves depression and manipulation of the cardiac and respiratory systems and the anesthesia team must be permitted to use all their skills to provide the best possible anesthetic outcome for the patient regardless of preoperative DNR status.	124 (32%)	145 (38%)	30 (8%)	62 (16%)	19 (5%)
Since patients do not have the knowledge to adequately appreciate the idiosyncrasies involved in the practice of medicine, physicians should independently evaluate what is in the best interest of patients regardless of the contents of an advance directive with regard to perioperative DNR status.	15 (4%)	38 (10%)	37 (10%)	165 (43%)	126 (33%)
DNR status should be respected during the intraoperative (while under the care of the anesthesia team to include the post anesthesia care unit) course because resuscitative issues are not the private preserve of health care providers but rather based on the patient’s own value system.	76 (20%)	113 (30%)	69 (18%)	107 (28%)	15 (4%)
DNR status should be disregarded during the perioperative phase of patient care because there is increased likelihood of successful resuscitation, regardless of the precipitating event, in the highly monitored setting of the operating room.	25 (7%)	96 (25%)	78 (20%)	139 (36%)	41 (11%)
Since it is difficult to distinguish between cardiorespiratory arrest that may occur spontaneously and that which occurs due to therapeutic intervention under anesthesia, DNR status should be disregarded during a patient’s perioperative course.	37 (10%)	126 (33%)	59 (15%)	126 (33%)	31 (8%)
If the patient has sufficient capacity to consent to the risks and benefits intrinsic to surgery and anesthesia, they have sufficient capacity to refuse or agree to attempts at resuscitation resulting from an intraoperative cardiopulmonary arrest.	130 (34%)	162 (42%)	40 (10%)	39 (10%)	9 (2%)

Concerning the technical aspect of resuscitative measures, almost half (47%) disagreed with a statement that DNR requests should be disregarded due to increased success of resuscitation perioperatively (Doctor Question 12). Fewer surgeons disagreed with this statement (p < 0.01). Of those who expressed an opinion, there was an equal split (51% and 49%, p = 0.74) between those who agreed and disagreed with a statement that DNR orders should be disregarded because of difficulty distinguishing between iatrogenic and spontaneous cardiorespiratory arrest (Doctor Question 13).

## Discussion

A majority of patients in our study had previously made a decision regarding perioperative resuscitation, with just over half confirming this in writing. Thus, a substantial number of anesthesiologists are expected to provide care to patients who have pre-existing DNR orders.

Nearly one-third (30%) of doctors in our study stated that they would automatically suspend a patient’s DNR order - lower than the 43% reported elsewhere in 1994 [20]. In a previous analysis, 60% of anesthesiologists, 37% of surgeons and 34% of internists assumed DNR suspension in the perioperative period [20]. Our findings suggest anesthesiologists’ views and practices have changed over 18 years, perhaps due to increased awareness of the published guidelines. A minority of anesthesiologists (18%) would automatically suspend a patient’s DNR order, whereas the rates of suspension by surgeons (38%) and internists (34%) in our cohort were similar to previous reports [20].

Responding to a fictional patient scenario designed to highlight the difficulties associated with preoperative DNR orders 53% stated they were unlikely to follow the order. A majority (64%) of those who would not follow the DNR order considered the non-contemporaneous nature of the DNR order to be of importance. Nevertheless, patients do not renounce their right to decide their care merely because they are entering an operating room [5]. Patients may prefer to have their DNR status retained for several reasons, including concerns regarding a worsened state following successful resuscitation, and that expiring under anesthesia is a more peaceful death [5].

Many doctors were concerned with risk of liability in either following or forgoing a patient’s DNR order during surgery, although risk consideration did not seem to affect decision-making. Others have suggested that a patient’s long- established right to refuse medical care, state statutes reflecting this right, and the small number of cases finding providers liable for following DNR orders contribute to limited risk for liability involving DNR orders during the perioperative period [21]. It should reassure doctors practicing in the United States that rarely will following a patient’s request for refusal of aggressive care given a properly addressed DNR order result in liability [16,21]. Most cases have involved conflicts with patient or surrogate informed consent to forego resuscitation [21]. However, when resuscitation occurs despite a DNR order, courts have been resistant to provide damages for continued or “wrongful life” [5,21].

The vast majority of doctors surveyed who would (86%) and would not (93%) comply with the patient’s DNR request were concerned that a pre-operative discussion about DNR status under anesthesia had not occurred. This level is higher than reported previously [22,23] where only approximately half of anesthesiologists in those studies stated they would discuss automatic DNR suspension with patients. In accordance with Joint Commission policy, our institution requires doctor-patient discussion of DNR orders prior to surgery [24]. One possible outcome includes continuation of DNR status. Given competing interests that accompany a patient with a DNR order to the OR, it is suggested that an automatic reconsideration of patient DNR status take place prior to surgical procedures [1,5,20,21]. An open discussion between patient and doctor may enable patients to better appreciate the likelihood of needing intraoperative resuscitation. Furthermore, this “required consideration” may better enable the health care providers to understand the patient’s wishes with regard to resuscitation efforts [1,5]. The majority of providers (77%) in our study believed patients can appreciate the idiosyncrasies involved with OR care and make decisions regarding resuscitation efforts. Furthermore, an equal percentage agreed that patients who have sufficient capacity to consent to surgery also have the capacity to refuse or agree to resuscitation efforts during the perioperative period. Our results reflect the importance of provider-patient discussions in informing patients about outcome of intraoperative resuscitation efforts while ensuring patient autonomy in decision-making.

Perhaps reflecting the continued frustration that doctors may feel when confronted with a patient who has a pre-existing DNR order is that 55% of those in our survey considered it illogical for a patient to undergo anesthesia in the presence of a DNR order. Commonly used anesthetic medications would cause death were it not for the anesthesiologist’s interventions. Doctors may believe that perioperative DNR orders limit providers from “sav[ing] their patients [5].” Seventy percent of those we surveyed agreed that anesthesiologists should be permitted to use their skills to provide best outcome for patients regardless of DNR status perhaps reflecting the increased likelihood of successful resuscitation in the OR [3,5,11-16]. Almost half (47%) of those doctors we surveyed agreed that DNR status should be suspended due to the increased likelihood of successful resuscitation in the OR environment. Although our survey did not assess whether doctors would forego offering surgical care to patients who refused to suspend their DNR orders perioperatively, a recent study reported that 54% of surgeons queried would refuse to operate on patients whose directives placed limits on postoperative care [25]. Since it may at times be difficult to distinguish between cardiopulmonary arrest and iatrogenic (anesthesia-related) depression of vital organs, some providers find it difficult to follow a patient’s DNR status intraoperatively [5,9]. However, an equal number of those doctors we surveyed agreed as disagreed with this concern.

Provider uncertainly and confusion when caring for patients with pre-existing DNR orders may be mitigated through better appreciation for patient expectations concerning their resuscitative care. Over half (57%) of patients felt that preoperative DNR orders should be suspended during anesthesia for an elective surgical case. Despite this, an overwhelming majority (92%) expected doctors to discuss their requests not to be resuscitated. These findings are similar to those reported by Clemency et al. when interviewing terminally ill patients with DNR orders [26]. Two matters appeared to be important to those surveyed by Clemency - “being ready to die” and limiting financial and emotional burdens to themselves and family members [26]. Upon learning how anesthesia care is provided and managed, some in Clemency’s study acknowledged that this was a different circumstance and temporarily suspended their DNR orders [26].

Over half (56%) of those patients we surveyed agreed that the type of surgery should influence whether DNR orders remained during the perioperative period and a large majority (79%) expected that if orders were suspended, they should be reinstated at a predetermined point postoperatively. These results are consistent with the work of others [26]. Our finding further reflect the importance of provider-patient communication given that patients may have different expectations for how their pre-existing DNR orders will be managed during the perioperative period.

The limitations of our study must be recognized. Our respondents were from a single center. Although our institution has a large referral base which is geographically and socio-economically diverse, whether the results can be extrapolated to other patient populations is uncertain. Tertiary referral centers such as ours employ doctors from diverse medical, geographical and cultural backgrounds. It is therefore possible that the diverse staff background may lead to a different mix of social, religious and ethical beliefs than would be seen in other medical centers, potentially limiting the external validity of the results. The patient survey response rate was excellent but the doctor response rate was suboptimal – especially from internal medicine physicians. Nonetheless we obtained a sample of almost 400 doctors. The differences between specialists may reflect a type II error because of non-comparable response rates between physician types. A real difference, assuming it exists, might also be a reflection of differences in awareness of the existence and content of perioperative DNR guidelines from specialist organizations rather than opinions formed on the basis of an individual’s specialty. Our survey assessed the importance patients and doctors place on communication, but we did not gauge the level of respondent appreciation for the necessity of both a thorough understanding of the resuscitative process and the need for bidirectional communication. Because “partial” resuscitation (e.g. chest compressions but no defibrillation) is not consistent with our medical center’s written DNR policy, and because DNR is compatible with maximal medical intervention, we did not ask our patients which resuscitative services would be acceptable in the OR. This policy may further limit the applicability of our results to other institutional practices. Our study did not ask patients to provide information concerning the extent of their upcoming surgical procedure or overall level of health. Both these factors may influence the desire to discuss resuscitation. Although our survey found that 54% of patients reported that they had previously documented their decision on resuscitation, the type of written form (DNR orders, power or attorney, living will) this took is unknown and unverified. Finally, because our study queried patients and providers from a single United States medical center, our results may not be applicable to other health care settings across the globe. Beliefs about the primacy of patient autonomy in the U.S and the legal system that helps to protect this right may be quite different from what exists elsewhere.

## Conclusions

In summary, active participation on the part of anesthesiologists, surgeons and internists will help ensure that the underlying aims of patients’ DNR orders are fulfilled during the perioperative period [26]. Although many of the patients we studied may agree that their DNR orders should be suspended perioperatively, this assumption is not universal and should not be presumed. The patients studied expected to be informed of how resuscitation efforts under anesthesia are, and are not, similar to those outside the OR environment. In this study anesthesiologists (versus surgeons and internists) were more likely to report that DNR orders should not be automatically suspended in the perioperative period.

## Abbreviations

ASA: American Society of Anesthesiologists; DNR: Do Not Resuscitate; OR: Operating room.

## Competing interests

Christopher M. Burkle, MD, JD, Keith M. Swetz, MD, MA, Matthew H. Armstrong, MD, MA and Mark T. Keegan, MB, MRCPI, MSc have no financial or non-financial competing interests.

## Authors’ contributions

CMB (MD, JD), KMS (MD, MA), MHA (MD, MA) and MTK (MB, MRCPI, MSc), Substantial contributions to: conception and design, data acquisition, analysis, and interpretation; Drafted and revised manuscript for important intellectual content. All authors read and approved the final manuscript.

## Pre-publication history

The pre-publication history for this paper can be accessed here:

http://www.biomedcentral.com/1471-2253/13/2/prepub

## Supplementary Material

Additional file 1 Patient Questionnaire. Click here for file

Additional file 2 Following or Suspending DNR Status in the Operating Room Environment: A survey assessment. Click here for file
