# Supplementary material for: Patient and doctor attitudes and beliefs concerning perioperative do not resuscitate orders: anesthesiologists’ growing compliance with patient autonomy and self determination guidelines
Source: BMC Anesthesiol. 2013 Jan 15;13:2. doi: 10.1186/1471-2253-13-2 (PMC3548687; doi:10.1186/1471-2253-13-2)
Supplement: Additional file 1 — Patient Questionnaire. [file 1471-2253-13-2-S1.pdf]

SURVEY NUMBER _____

INSTRUCTIONS:
Please check the box that best corresponds to your answer for each question below. Your questionnaire sheet will be collected with your other materials by the clinic staff. Thank you for your willingness to assist us with this project.

(1) Have you made a decision as to whether you wish to be resuscitated (e.g. chest compressions, electrical shock, emergency medications) in the event that you fall seriously ill?

☐ Yes
☐ No
☐ Prefer not to answer

(2) If you have made a decision as to whether you wish to be resuscitated, have you documented your wishes in written form?

☐ Yes
☐ No
☐ Prefer not to answer

PLEASE INDICATE YOUR LEVEL OF AGREEMENT OR DISAGREEMENT WITH THE FOLLOWING STATEMENTS:

	**Strongly Agree**	**Somewhat Agree**	**Neither Agree or Disagree**	**Somewhat Disagree**	**Strongly Disagree**	**Prefer not to answer**
(3) If a patient has determined that they wish not to be resuscitated should they fall ill, this request should be automatically suspended while they undergo a surgical procedure.	☐	☐	☐	☐	☐	☐
4) The request not to be resuscitated should always be discussed between the patient and surgeon or anesthesiologist prior to undergoing surgery.	☐	☐	☐	☐	☐	☐
(5) If a patient's request not to be resuscitated is suspended for the length of surgery, it should be reinstated at a predetermined point following the completion of the surgical procedure.	☐	☐	☐	☐	☐	☐

(6) The decision as to whether to resuscitate a patient during surgery should be left up to the surgeon and anesthesiologist alone because patients can not fully understand the complex processes that take place during a surgical procedure.	☐	☐	☐	☐	☐	☐
(7) The type of surgical procedure a patient undergoes should influence whether a patient's request not to be resuscitated is followed.	☐	☐	☐	☐	☐	☐

(8) What is your age (yr)?

☐ 21-35

☐ 36-50

☐ 51-65

☐ 66-70

☐ >70

☐ Prefer not to answer

THANK YOU

This sheet will be collected with your other materials by the Clinic staff.
