# Supplementary material for: Patient and doctor attitudes and beliefs concerning perioperative do not resuscitate orders: anesthesiologists’ growing compliance with patient autonomy and self determination guidelines
Source: BMC Anesthesiol. 2013 Jan 15;13:2. doi: 10.1186/1471-2253-13-2 (PMC3548687; doi:10.1186/1471-2253-13-2)
Supplement: Additional file 2 — Following or Suspending DNR Status in the Operating Room Environment: A survey assessment. [file 1471-2253-13-2-S2.pdf]

Honoring DNR Status in the Operating Room Environment: A survey assessment

I. Demographics

1) Please indicate your area of primary practice:

- ☐ Surgery
- ☐ Internal Medicine (primary/general focus)
- ☐ Internal Medicine (subspecialty focus)
- ☐ Anesthesiology
- ☐ Other
- ☐ Prefer not to answer

2) Please indicate your practice level:

- ☐ Consultant with 0-3 years of experience
- ☐ Consultant with 4-7 years of experience
- ☐ Consultant with 8-15 years of experience
- ☐ Consultant with 16 or more years of experience
- ☐ Fellow
- ☐ Resident
- ☐ Prefer not to answer

II. Please answer the single question listed below:

3) A patient's advance directive stating refusal of CPR and advanced resuscitative procedures is automatically revoked for the duration of the patient's intraoperative (while under the direct care of the anesthesia team to include the post anesthesia care unit) course.

- ☐ True
- ☐ False
- ☐ Unsure
- ☐ Prefer not to answer

III. Please answer the questions related to the patient scenario listed below:

A 65 year married female is to undergo a mastectomy for biopsy proven breast cancer. Her mother has resided in a skilled nursing facility for ten years since suffering a cardiac arrest following repair of an abdominal aortic aneurysm. Seven years ago, the patient's father suffered a massive CVA (cerebral vascular accident) causing him to be bed ridden and requiring tube feedings until his subsequent death two years later. During her first visit to the clinic five years ago for a general medical exam, the patient signaled her request via an advance directive that CPR (cardiopulmonary resuscitation) and advanced resuscitation procedures be withheld in the event of a cardiac or respiratory arrest. Documentation that the patient has an advance directive on file was placed in the electronic patient record by the perioperative nursing staff.

4) How likely would you be to honor the advance directive form and not provide CPR and advanced resuscitative procedures during the patient's intraoperative (while under the direct care of the anesthesia team to include the post anesthesia care unit) course?

- ☐ Very Likely
- ☐ Somewhat Likely
- ☐ Unsure
- ☐ Somewhat Unlikely
- ☐ Very Unlikely
- ☐ Prefer not to answer

Please assign relative value to the items listed below as to the importance they played in your ultimate decision to follow or not follow the patient's advance directive.

5) Initial signing of advance directive was five years ago during a general medical exam visit.

- ☐ Very Unimportant
- ☐ Unimportant
- ☐ Neither Important or Unimportant
- ☐ Important
- ☐ Very Important
- ☐ Prefer not to answer

6) Fear of legal liability if advance directive is honored or not honored.

- ☐ Very Unimportant
- ☐ Unimportant
- ☐ Neither Important or Unimportant
- ☐ Important
- ☐ Very Important
- ☐ Prefer not to answer

7) A discussion with the patient regarding resuscitation had not occurred during the pre-operative visit or in the outpatient setting at the time of surgical listing.

- ☐ Very Unimportant
- ☐ Unimportant
- ☐ Neither Important or Unimportant
- ☐ Important
- ☐ Very Important
- ☐ Prefer not to answer

IV. Please indicate your level of agreement or disagreement with the following statements UNRELATED to the previous patient scenario:

8) Since anesthesia for surgery basically represents a resuscitative effort (endotracheal intubation, pressor support, etc.), DNR ("Do not Resuscitate") status makes no logical sense in the context of a surgical procedure requiring an anesthetic.

- ☐ Strongly Disagree
- ☐ Disagree
- ☐ Neither Agree or Disagree
- ☐ Agree
- ☐ Strongly Agree
- ☐ Prefer not to answer

9) The delivery of every anesthetic likely involves depression and manipulation of the cardiac and respiratory systems and the anesthesia team must be permitted to use all their skills to provide the best possible anesthetic outcome for the patient regardless of preoperative DNR status.

- ☐ Strongly Disagree
- ☐ Disagree
- ☐ Neither Agree or Disagree
- ☐ Agree
- ☐ Strongly Agree
- ☐ Prefer not to answer

10) Since patients do not have the knowledge to adequately appreciate the idiosyncrasies involved in the practice of medicine, physicians should independently evaluate what is in the best interest of patients regardless of the contents of an advance directive with regard to perioperative DNR status.

- ☐ Strongly Disagree
- ☐ Disagree
- ☐ Neither Agree or Disagree
- ☐ Agree
- ☐ Strongly Agree
- ☐ Prefer not to answer

11) DNR status should be respected during the intraoperative (while under the care of the anesthesia team to include the post anesthesia care unit) course because resuscitative issues are not the private preserve of health care providers but rather based on the patient's own value system.

- ☐ Strongly Disagree
- ☐ Disagree
- ☐ Neither Agree or Disagree
- ☐ Agree
- ☐ Strongly Agree
- ☐ Prefer not to answer

12) DNR status should be disregarded during the perioperative phase of patient care because there is increased likelihood of successful resuscitation, regardless of the precipitating event, in the highly monitored setting of the operating room.

- ☐ Strongly Disagree
- ☐ Disagree
- ☐ Neither Agree or Disagree
- ☐ Agree
- ☐ Strongly Agree
- ☐ Prefer not to answer

13) Since it is difficult to distinguish between cardiorespiratory arrest that may occur spontaneously and that which occurs due to therapeutic intervention under anesthesia, DNR status should be disregarded during a patient's perioperative course.

- ☐ Strongly Disagree
- ☐ Disagree
- ☐ Neither Agree or Disagree
- ☐ Agree
- ☐ Strongly Agree
- ☐ Prefer not to answer

14) If the patient has sufficient capacity to consent to the risks and benefits intrinsic to surgery and anesthesia, they have sufficient capacity to refuse or agree to attempts at resuscitation resulting from an intraoperative cardiopulmonary arrest.

- ☐ Strongly Disagree
- ☐ Disagree
- ☐ Neither Agree or Disagree
- ☐ Agree
- ☐ Strongly Agree
- ☐ Prefer not to answer
